# Standardization of a new non-invasive device for assessment of arterial stiffness in rats: Correlation with age-related arteries’ structure

**DOI:** 10.1016/j.mex.2020.100901

**Published:** 2020-04-27

**Authors:** Mayara F. Fabricio, Maria T. Jordão, Danyelle S. Miotto, Thalles F.R. Ruiz, Carlos A. Vicentini, Silvia Lacchini, Carlos Ferreira Santos, Lisete C. Michelini, Sandra L. Amaral

**Affiliations:** a Joint Graduate Program in Physiological Sciences, PIPGCF, UFSCar/UNESP, São Carlos, SP, Brazil; b Department of Physical Education - São Paulo State University (UNESP), School of Sciences, Bauru, SP, Brazil; c Department of Biological Sciences, São Paulo State University (UNESP), School of Sciences, Bauru, SP, Brazil; d Department of Physiology and Biophysics, Biomedical Sciences Institute, University of São Paulo (SP), Brazil; e Department of Anatomy, Biomedical Sciences Institute, University of São Paulo (SP), Brazil; f Bauru School of Dentistry, University of São Paulo, Bauru, SP, Brazil

**Keywords:** Pulse wave velocity, Aging, Hypertension, Aorta, Carotid, Femoral, Collagen deposition

## Abstract

Pulse wave velocity (PWV) has become a gold standard index to quantify the stiffness of the aorta and is a predictor of cardiovascular events. A recent paper compared the pOpmètre^Ⓡ^, a device for measuring the finger-toe PWV, with other techniques and demonstrated its accuracy and validity. However, human devices do not allow the advancement of our knowledge on conditioning mechanisms. Based on its human validation, a new device, pOpet^Ⓡ^ 1.0 system was designed for estimation of PWV in small animals and this present study aimed to standardize the pOpet^Ⓡ^ 1.0 for estimation of arterial stiffness in rats, and to confirm its liability and stability as well as the reproducibility of assessments. Therefore several precautions were taken into consideration like as the correct position of the animal and photodiodes according to manufacturers’ suggestions. Results indicated that estimation of PWV through the new pOpet^Ⓡ^ 1.0 device exhibits good internal consistency, stability and objectivity in all tests performed between days and evaluators. Importantly, data suggest for the first time that this new device is able to detect changes in arterial stiffness that are conditioned by age and pressure-related arterial remodeling.

• This new pOpet^Ⓡ^ device is able to detect changes in vessel structure.

• This new pOpet^Ⓡ^ device exhibits good internal consistency, stability and objectivity in all tests performed

• Correct position of the animal and photodiodes are crucial to obtain a very stable signal.

## Graphical abstract

**Figure:**
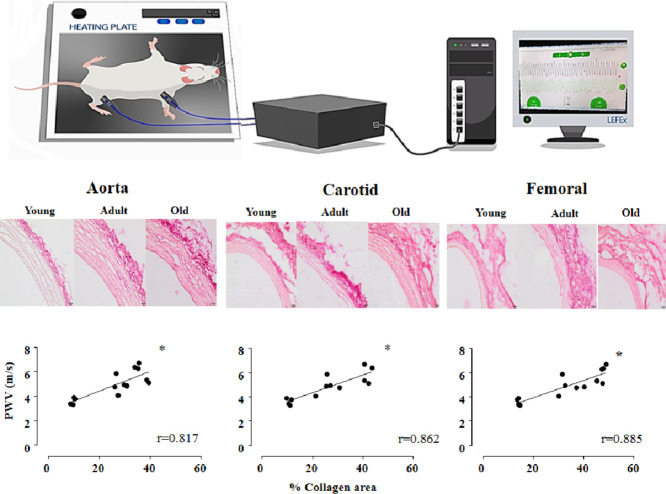

**Table:** Specifications Table

Subject Area:	Medicine and Dentistry
More specific subject area:	*Cardiovascular Physiology*
Method name:	*pOpet^Ⓡ^ 1.0 system for small animals*
Name and reference of original method:	*ALIVON, M., VO-DUC PHUONG, T., VIGNON, V., BOZEC, E., KHETTAB, H., HANON, O., BRIET, M., HALIMI, J. M., HALLAB, M., PLICHART, M., MOHAMMEDI, K., MARRE, M., BOUTOUYRIE, P. & LAURENT, S. 2015. A novel device for measuring arterial stiffness using finger-toe pulse wave velocity: Validation study of the pOpmetre(R). Arch Cardiovasc Dis, 108, 227-34.*
Resource availability:	*pOpet^Ⓡ^ 1.0 system for small animals (Axelife SAS, Saint Nicolas de Redon, France)*

## Methods

Epidemiological and longitudinal studies reveal that measurement of aortic stiffness has a superior predictive value for determining the risk of cardiovascular diseases compared to classical risk factors and, as such, has emerged as an important “biomarker” predictive of end-organ damage and overall cardiovascular risk [1], [2], [3]. Indeed, the evaluation of PWV along the arterial network has become a gold standard index to quantify the stiffness of the aorta [4] and several techniques were thought to evaluate the distensibility/compliance of the vessels [5]. Recently, a novel device for measuring the finger-toe PWV, pOpmètre^Ⓡ^, was validated and demonstrated accuracy and validity for arterial stiffness measurement along with some advantages like simplicity, rapidity, feasibility, patient acceptability besides the good agreement with reference techniques [6]. However, this device was available for humans, not for experimental animals. Based on its human validation and in the lack of a good technique to uncover the mechanism conditioning changes in arterial stiffness, a pOpet^Ⓡ^1.0 for animal studies was created. However, this device has not been validated for small animals. Therefore, in this study we aimed to standardize the pOpet^Ⓡ^ for estimation of arterial stiffness in rats, and to confirm its liability and stability as well as the reproducibility of assessments. Knowing that vessel compliance is an aging-related phenomenon [5], [7], [8], [9], [10], [11] we estimated in rats of different ages the PWV and analyzed their arteries’ structure and the content of its constituents. In addition, to check the ability of pOpet^Ⓡ^1.0 to precisely detect different arterial stiffness levels, we compared PWV in hypertensive and normotensive rats. Therefore, for the standardization of this technique 3 studies were performed.

## Methods details

All rats were obtained from Center for Research and Production Facilities of UNESP (Botucatu, SP, Brazil). This study was conducted in accordance with Guidelines for the use of experimental animals and was approved by the Ethical Committee on Animal Experiments of the School of Sciences in Bauru, São Paulo State University – UNESP (#1098/2016 Vol.1). All animals were housed in an animal facility with controlled-temperature room (22 ± 2°C), with a 12/12 h light/dark cycle. Food and water were provided *at libitum*. Body weight was measured in conscious animals before anesthesia.

**Study 1:** This study was designed to standardize the pOpet^Ⓡ^ 1.0 system for estimation of PWV in small animals in order to give support for researchers interested in conditioning mechanisms of arterial stiffness. For this study, 6 male Wistar rats (~300 g) were used.

**Fig. 1:**
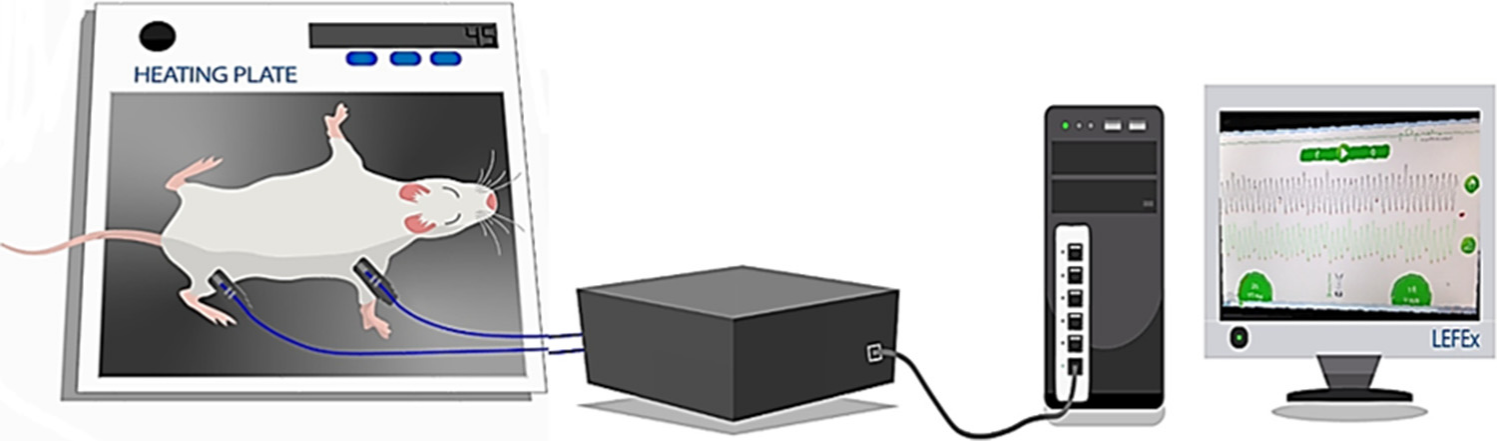
Image illustrating the correct positioning of rat and the two photodiodes: one on right forearm, close to elbow and another one on hindlimb, close to the knee on anesthetized rat, placed on heating plate. Photodiodes are connected to a computer and transit time (TT, ms) between probes (carotid-femoral path) can be detected by pOpet 1.0 software, which calculates pulse wave velocity (PWV, m/s). Distance between photodiodes is included in the software before each animal analysis, for calibration. See animal and room conditions in Methods details section.

Since this device was commercialized based on its humans study, we provide in this article some important information about correct position of the animal and photodiodes followed by several precautions, which are crucial to obtain a very stable signal. Therefore, rats were anesthetized with an intraperitoneal injection of xylazine hydrochloride (10 mg/kg) and ketamine hydrochloride (50 mg/kg, Vetbrands). Two pOpet^Ⓡ^ probes (Axelife SAS, Saint Nicolas de Redon, France) were positioned on the right forelimb (close to elbow) and hindlimb (close to knee) members for PWV assessment, as shown in Fig. 1. According to the manufacturer, this probes’ positioning, allowing the subtraction of the time between heart to knee minus heart to elbow, will give the aortic path. Therefore, the pOpet noninvasive device will measure the transit time from the 2 probes. The Photo Diode (PD) part of the pOpet^Ⓡ^ probes, that is the reception of the signal, was positioned on the top of the forelimb /hindlimb, and the other part, the infrared light (LED), on the bottom. The travelled distance of the pulse wave was estimated in each rat by the distance (D, cm) between the two photodiodes (measured with tape). To get a good signal, we kept the temperature of the room (22° C) and heating plate (43-45°C) as stable as possible and did not use a very intense light. Important to note that rat´s temperature was maintained at 37 ± 1°C. In a quiet room, after stabilization of the signal, heating pad was turned off and transit time (TT, ms) were recorded during 10 seconds and registered by pOpet 1.0 software (Fig. 1). Taking together the travelled distance (D) and transit time (TT), we can calculate PWV using the following formula: PWV_(m/s)_ = D_(m)_/TT_(s)_, as published [12]. For all studies, 10 measurements of each rat were done and the average was calculated

In order to check the accuracy of measurements, after the above procedure, we performed an invasive study in which the rat (n=2) had its carotid and femoral arteries catheterized. Then, from arterial pressure recordings (at the same time), we could calculate the delta time between pressure pulses from carotid and femoral arteries. It was found that values of TT (ms), given by pOpet noninvasive device, were equal to delta time (s) obtained from invasive arterial pressure measurement. After that, under anesthesia overload, rat was euthanized and the size of its aorta and iliac arteries was measured. This value was equal to distance between probes that we measure with tape in order to calibrate the system.

**Fig. 2:**
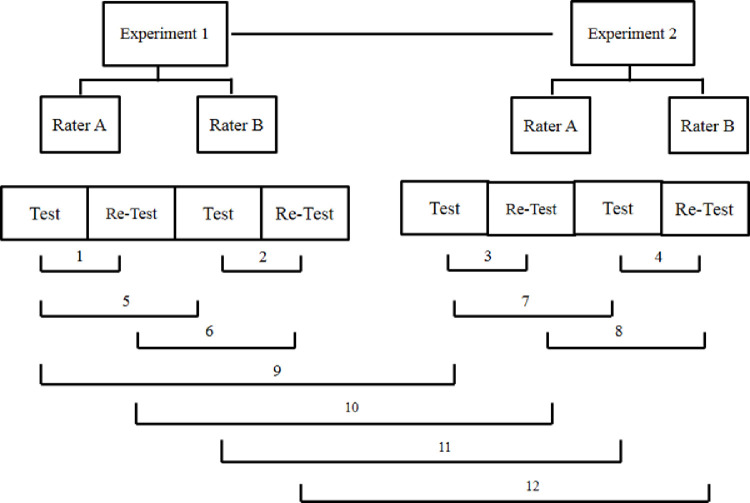
Test and re-test schedule for internal consistency, stability and objectivity analyses. **Experiment** 1, first day of measurement; **Experiment 2**, second day of measurement; **rater A** (evaluator A); **rater B** (evaluator B); test: first measurement on each day for each evaluator; re-test: second measurement (after reconnection of device) on each day for each evaluator. Sequence of tests: **1**: test-retest of rater A on **Experiment 1**; **2**: test-retest of rater B on **Experiment 1**; **3**: test-retest of rater A on **Experiment 2**; **4**: test-retest of rater B on **Experiment 2**; **5**: Comparison of first measurement (test) between raters A and B on **Experiment** 1; **6**: Comparison of second measurement (re-test) between raters A and B on **Experiment 1**; **7**: Comparison of first measurement (test) between raters A and B on **Experiment 2**; **8**: Comparison of second measurement (re-test) between raters A and B on **Experiment 2**; **9**: comparison of test results of rater A between **Experiment 1** and **Experiment 2**; **10**: comparison of re-test results of rater A between **Experiment 1** and **Experiment 2**; **11**: comparison of test results of rater B between **Experiment 1** and **Experiment 2**; **12**: comparison of re-test results of rater B between **Experiment 1** and **Experiment 2.**

In order to test internal consistency, objectivity and stability of the PWV estimations, we performed and repeated several tests according the schedule shown in Fig. 2. Different sessions were represented by **test**and **re-test**; different days were indicated by **Experiment 1** and **Experiment 2** and different evaluators by **rater A** and **rater B**. To test the internal consistency (same-day test-retest), the researcher performed 10 measurements, removed both photodiodes and disconnected the equipment from power. After 2 minutes, the researcher reconnected the photodiodes and the equipment in the same rat and performed another 10 measurements (1, 2, Fig. 2). This experiment was repeated on the next day (3, 4, Fig. 2).

To test for objectivity (Inter-rater reliability - across different researchers), two researchers (Rater A and B) performed the same procedures and their results were compared to each other. This test was carried out during both Experiment 1 (5 and 6, Fig. 2) and experiment 2 (7 and 8, Fig. 2).

To test the pOpet^Ⓡ^ 1.0 stability (test-retest on different days), all measurements were made again on the next day (1-day apart) by the same researchers (9 and 10 for Rater A; 11 and 12 for Rater B).

To evaluate the hypothesis that the sample means values did not differ after a test and retest (internal consistency, stability and objectivity), a paired student t-test was used. Likewise, the results were further confirmed using the Intraclass Correlation Coefficient – ICC test (two-way mixed, consistency, average-measures ICC) to determine the level of reproducibility between measurements on 2 sessions performed at the same day, at different days and by different raters [13,14]. ICC values (Cronbach´s α) interpretation of the reported values for each ICC were based upon the following criteria: <0.50 = poor, 0.50 - 0.75 = moderate, 0.75 – 0.9 = good and >0.9 = excellent, as used by [14]. Therefore, values greater than or equal to 0.80 were considered to show very good reproducibility between test and retest measurements and between raters (α < 0.05). As observed in Table 1, most of the ICC results **(**Cronbach´s alpha value) for different combinations was higher than 0.9 (only two comparisons were between 0.8-0.9) which means a good to excellent reliability for the PWV estimations (average of ICC= 0.929). Table 2 shows the results of comparisons between raters (student T- test). It is possible to note that none of the comparisons attained a significant difference (T-test, α>0.05). These results gave us assurance that values were consistent and, therefore, we suggest that this technique provides a stable and valuable estimation of PWV.

**Table 1:** Results of Intraclass Correlation Coefficient (ICC) tests: internal consistency (test-retest in the same day), stability (test-retest on different days)

Tests	experiments	rater A	rater B
test x re-test	1	0.994 (1)	0.945 (2)
test x re-test	2	0.986 (3)	0.969 (4)
test x test	1 × 2	0.949 (9)	0.877 (10)
re-test x re-test	1 × 2	0.904 (11)	0.808 (12)

Results are Cronbach´s Alpha for Intraclass Correlation Coefficient (ICC) tests. Numbers in the parenthesis are the sequence of tests following Fig. 2:

1: test-retest of rater A on Experiment 1;

2: test-retest of rater B on Experiment 1;

3: test-retest of rater A on Experiment 2;

4: test-retest of rater B on Experiment 2;

9: comparison of test results of rater A between Experiment 1 and Experiment 2;

10: comparison of re-test results of rater A between Experiment 1 and Experiment 2;

11: comparison of test results of rater B between Experiment 1 and Experiment 2;

12: comparison of re-test results of rater B between Experiment 1 and Experiment 2.

**Table 2:** Results of Student-T test between raters: objectivity (inter-rater reliability)

Tests	experiments	Rater A x rater B
test x test	1	0.6443 (5)
re-test x re-test	1	0.4634 (6)
test x test	2	0.6619 (7)
re-test x re-test	2	0.4679 (8)

**Fig. 3:**
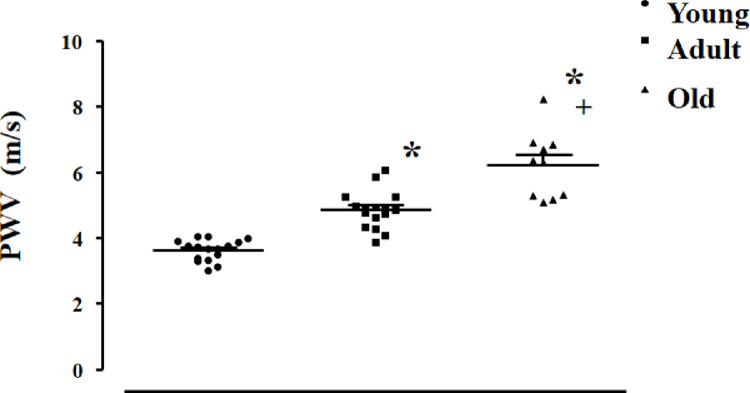
Estimated values of pulse wave velocity (PWV, m/s) in all analyzed groups: young (n = 16), adult (n = 16) and old (n = 10). Transit time was 17.65 ± 0.40 ms, 21.9 ± 0.66 ms and 18.93 ± 0.93 ms for young, adult and old rats, respectively. Distance between probes 6.38 ±0.13cm, 10.5 ± 0.11 cm and 11.5 ± 0.13 cm, for young, adult and old rats, respectively. Significance: One way ANOVA test with Tukey as post-hoc: * vs young, **+** vs adult, p <0.05

Results are p values of student-t tests. Numbers in the parenthesis are the sequence of tests following Fig. 2:

5: Comparison of first measurement (test) between raters A and B on Experiment 1;

6: Comparison of second measurement (re-test) between raters A and B on Experiment 1;

7: Comparison of first measurement (test) between raters A and B on Experiment 2;

8: Comparison of second measurement (re-test) between raters A and B on Experiment 2;

Study 2: This study aimed to correlate the estimated value of PWV by pOpet^Ⓡ^ 1.0 with age-induced changes in vessels morphology (and stiffness). For this, PWV was assessed in young, adult and old normotensive rats and correlated with age-induced morphometric changes in different arteries. For this study, 42 male Wistar rats of different ages were used and allocated into 3 groups: Young: 1 month (~100 g, n=16); Adult: 4 months (300-350 g, n=16) and Old: 12 months (~500g, n=10). As expected, body weight was different between groups, (81 ± 1.83g, 424 ± 10.89g and 566 ± 15.06 g, for young, adult and old groups, respectively). Fig. 3 shows that estimated PWV values were smaller in young rats and increased from young to adult and from adult to aged rats (3.63 ± 0.08 m/s, 4.86 ± 0.15 m/s and, 6.21 ± 0.32 m/s for young, adult and old groups, respectively, p<0.0001).

Twenty-four hours after PWV assessment, rats were deeply anesthetized with xylazine hydrochloride (20 mg/kg) and ketamine hydrochloride (160 mg/kg, Vetbrands). Immediately after respiratory arrest, a ventral incision was made in the midline of the thoracic region to expose the heart. Rats were submitted to transcardiac perfusion with ~100 ml of sterile saline followed by ~200 ml of 4% paraformaldehyde in buffered PBS, using a peristaltic Milan pump, at 20-30mL/min rate [15]. Segments (~5 mm) of descending thoracic aorta, femoral artery and common carotid artery were collected for smooth muscle, elastic and collagen tissue analyses (5 rats of each group).

For morphological, smooth muscle and elastic tissue analyses, vessel samples were cleaned and fixed for 24 h in 4% buffered paraformaldehyde solution in 0.1 mol/L phosphate buffer, pH 7.2. Samples were then dehydrated in graded ethanol concentrations (70, 80, 90 and 100%) and embedded in histological paraplast, as previously described [15]. Serial transverse sections (microtome, 10 µm thick sections) were obtained from each artery; 10 to 15 sections were mounted on glass slide and stained with hematoxylin and eosin (smooth muscle constituents) or Weigert-hematoxylin (elastic lamellas/ fibrils). Transverse sections of thoracic aorta (40x magnification) as well as carotid and femoral arteries (100x magnification) were examined with a microscope (Leica DM4 B); images were captured using a Leica MC170 HD camera coupled to the microscope. Morphometric analyses were performed off-line using ImageJ software. From each vessel, cross-sectional area (CSA, µm^2^), inner (ID, µm) and outer diameter (OD, µm) and internal radius were obtained. From these values, wall thickness [(OD–ID)/2, µm] and wall/lumen ratio (wall thickness/ID) were calculated as previously published [16]. Vessel wall CSA (µm^2^) was also calculated (outer CSA – inner CSA).

**Fig. 4:**
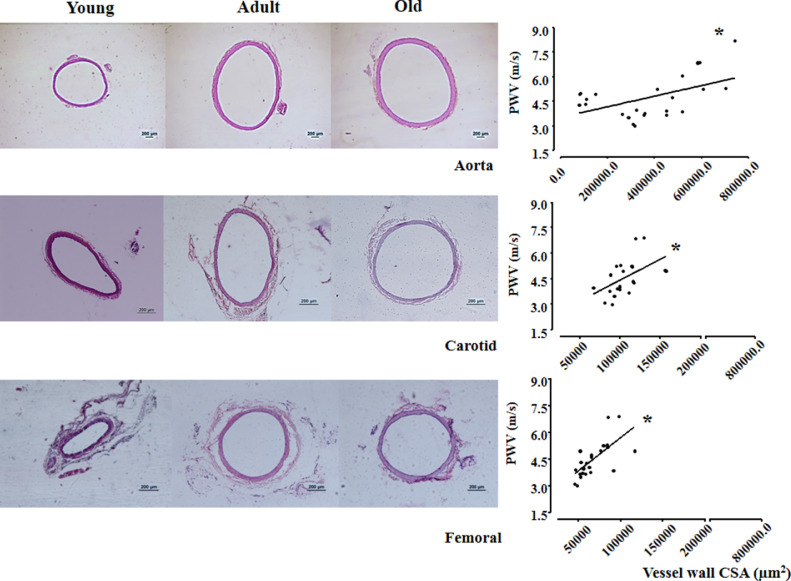
Left panel: Histological cross-sectional photos (10 µm) stained with hematoxylin and eosin, obtained from fragments of aorta (upper), carotid (middle) and femoral (bottom) arteries from 1 rat of each group (young, adult and old). Bars represent 200µm (objective: 40x for aorta artery and 100x for carotid and femoral arteries). Right panel: Pearson correlation between vessel wall cross-sectional area (CSA, µm^2^) of aorta (r=0.508. p=0.0113), carotid (r=0.519, p=0.0158) and femoral (r=0.683, p=0.0002) arteries. Significance: * p<0.05

Representative images of aorta, carotid and femoral arteries of young, adult and old rats are shown in Fig. 4. Hematoxylin-eosin staining depicted vessel layers within the 3 arteries in which we measured vessel wall CSA as well as the total CSA, including the luminal CSA. When compared with respective young arteries, vessel wall CSA increased with ageing (+96% in old aorta; +29% in the adult and old carotid; +36% in adult and +63% in the old femoral, Table 3). The ability of pOpet device to correctly detect changes in arteries’ morphometry was tested by Pearson correlations between PWV estimations and respective vessel morphometry (which impact vessel stiffness) in young, adult and old normotensive rats. Interestingly, in the three arteries (right panels in Fig. 4) the age-induced increase in vessel wall CSA was accompanied by significant increases in PWV values: positive correlations between the vessel wall CSA and PWV value were observed for the aorta (r=0.508, p=0.0113), carotid (r=0.519, p=0.0158) and femoral arteries (r=0.683, p=0.0002) among rats with different ages.

**Table 3:** Morphometric analysis of thoracic aorta, carotid and femoral arteries in the young, adult and old groups.

	ID (µm)	OD (µm)	wall thickness (µm)	wall-to-lumen ratio	Vessel wall CSA (µm^2^)	Total CSA (µm²)
aorta						
Young	1,214 ± 28	1,382 ± 34	84 ± 3	0.069 ± 0.002	345,750 ± 22,238	1,508,179 ± 75,359
Adult	1,165 ± 165	1,292 ± 180	63 ± 7	0.057 ± 0.002*	284,450 ± 65,422	1,565,225 ± 381,276
Old	1,932 ± 23*^+^	2,143 ± 28*^+^	106 ± 5^+^	0.055 ± 0.003*	676,488 ± 37597*^+^	3,609,288 ± 95,068*^+^
carotid						
Young	587 ± 15	678 ± 17	45 ±2	0.077 ± 0.002	90,493 ± 4,352	362,313 ± 17853
Adult	755 ± 14*	848 ± 14*	46 ± 3	0.062 ± 0.001	116,438 ± 7,878*	566,127 ± 19,057*
Old	883 ± 37*^+^	963 ± 37*^+^	40 ± 1	0.045 ± 0.002*^+^	116,098 ± 6,011	732,094 ± 57,314*^+^
femoral						
Young	394 ± 18	474 ± 14	40 ± 2	0.106 ± 0.011	53,870 ± 2,038	178,350 ± 10,525
Adult	530 ± 35*	603 ± 37*	36 ± 2	0.070 ± 0.004*	73,252 ± 5,655*	326,156 ± 30,878*
Old	606±16*	691±16*	43±1	0.071 ±0.02	87,750±2,938*	377,876 ± 18,036*

Values in mean ± SEM. Results of morphometric analysis on thoracic aorta (40x), carotid and femoral arteries (100x). ID = internal diameter; OD = outer diameter; wall = wall thickness; CSA = cross-sectional area in all experimental groups. Significance:  One way ANOVA test with Tukey as post hoc: * vs young ^+^*vs* adult, p <0.05.

In these arteries the total CSA (including lumen CSA) also increased with ageing (+139% in old aorta; +56% in adult and +102% in old carotid; +83% in adult and +113% in old femoral, Table 3). Because of the age-induced increase on external and internal diameter and the almost similar wall thickness (a small 20% increase was observed only in the old aorta), the wall-to-lumen ratio was significantly reduced with advanced age (on average -20% in the old aorta; -42% in the old carotid and -34% in adult and old femoral, Table 3).

**Fig. 5:**
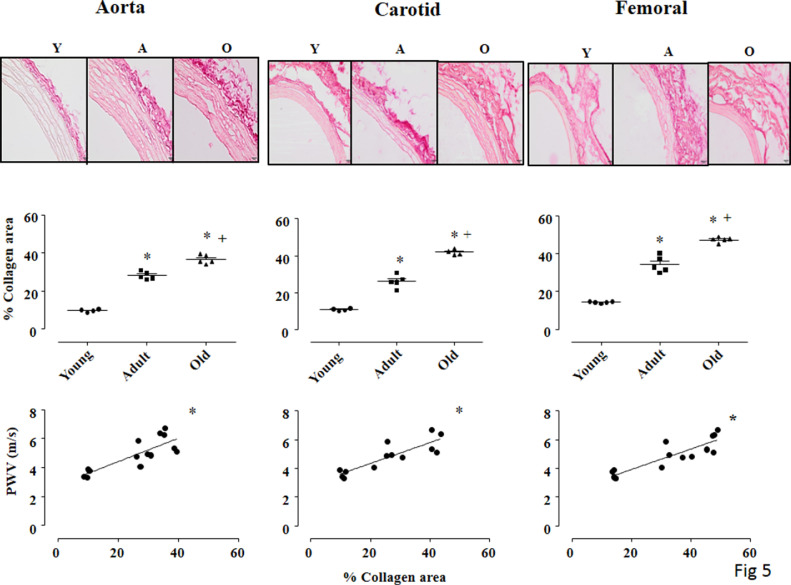
Upper panel: Picrosirius red-stained cross-sections of thoracic aorta (left panel), carotid (center panel) and femoral (right panel) arteries of all groups: young (Y), adult (A) and old (O) viewed under light microscope to show collagen fibers. 400 x magnification, Bar= 200µm. Middle panel: Values of % of collagen deposition area on transverse sections of thoracic aorta (left panel), carotid (center panel) and femoral (right panel) arteries. Bottom panel: Pearson correlation between % of collagen deposition area and estimated pulse wave velocity (PWV, m/s) in picrosirius-stained aorta (left panel, r=0.817, p=0.0004), carotid (center panel, r=0.862, p=0.0002) and femoral (right panel, r=0.885, p<0.0001) arteries among all rats. Significance: One way ANOVA test with Tukey as post hoc: * vs young, + *vs* adult, p<0.001

Knowing that collagen content is an important component to determine vessels’ stiffness [17], we also analyzed the collagen deposition area (%) in Picrosirius-red stained sections of aorta, carotid and femoral in young, adult and old rats (upper panel in Fig. 5). For collagen deposition area analysis, fresh segments of thoracic aorta, carotid and femoral arteries were embedded in Tissue-Tek O.C.T. (Sakura^Ⓡ^) and immediately frozen in liquid nitrogen. Histological sections of 5 µm were cut in the cryostat, mounted into slides and stained with Picrosirius Red (0.1% w/v), as previously described [18]. Bright field images for all arteries were analyzed by a light microscope (Olympus Bx43-SC30, 400x magnification). From each artery image, 4 different areas (same size) were analyzed, and the mean was calculated per animal. With the observer blinded to group identity, collagen deposition area (%) was identified and quantified by the image analysis software ImageJ.

Even though this technique (bright field images) does not allow a precise collagen quantity (limitation of this study), we can observe in all arteries a significant increase in collagen deposition area in adult, compared with young rats (on average + 160%) that was even greater in old rats (on average +43% *vs* adult, middle panels of Fig. 5). Again, strong positive correlations were observed between collagen deposition area (% of area) in the aorta and PWV estimated values (r=0.817, p=0.0004), carotid (r= 0.862, p=0.0002) and femoral (r=0.885, p<0.0001) arteries (bottom panels in Fig. 5).

**Fig. 6:**
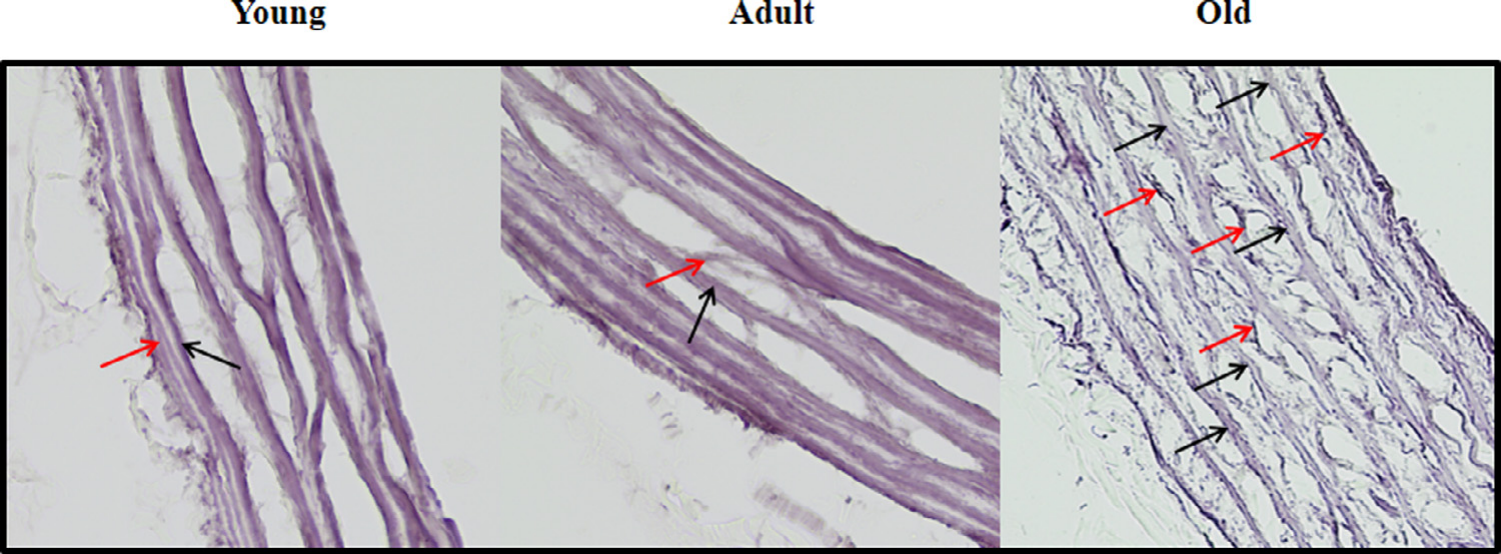
Elastic fiber networks in transverse sections of thoracic aorta (400x magnification) after Weigert-hematoxylin staining. Red arrow mean thin elastic fiber and black arrow means thicker elastic fibers. Both young and adult aorta show oriented thinner (red arrow) and thicker (black arrow) elastic fibers. Elastin disarray was obvious in aorta sections of older rats with presence of thinner elastic fibers probably from rupture of thicker fibers or remodeling.

It is also known that disruption of elastic fibers could contribute to augment vessels’ stiffness [17]. Therefore, we also analyzed the elastic fiber networks in transverse sections of thoracic aorta after Weigert-hematoxylin staining (400x magnification, Fig. 6). The disarray of elastin networks in the medial layer of aorta (graded in a scale of 1-4 levels) was determined by the loss of parallel orientation and increased fibers’ rupture. Level 1 reflects almost all elastin fibers aligned in parallel throughout the vascular wall, with almost no ruptures or ≤ 25%; Level 2 indicates distorted elastin fibers and few ruptures (26-50%); Level 3 reflects samples with more than a half of the elastin fibers disrupted (56- 75%) and level 4 indicates that almost all fibers were disrupted (≥ 76%). For this analysis, 3 different microscopic fields/rat were quantified at 400 × magnification. As shown in Fig. 6, both young and adult thoracic aortas show well-oriented thinner (red arrow) and thicker (black arrow) elastic fibers, with almost no disruptions (scores: 1.86 ± 0.23 and 1.93 ± 0.35, for young and adult rats, respectively). However, a severe elastin disarray pattern was observed in the aorta of older rats, with the presence of thinner elastic fibers probably originated from rupture of thicker fibers and/or elastin remodeling (score: 3.39 ± 0.25, p<0.05 *vs* younger and adult, using one way ANOVA test); this disarray could contribute to increase the aorta stiffness in aged rats.

**Fig. 7:**
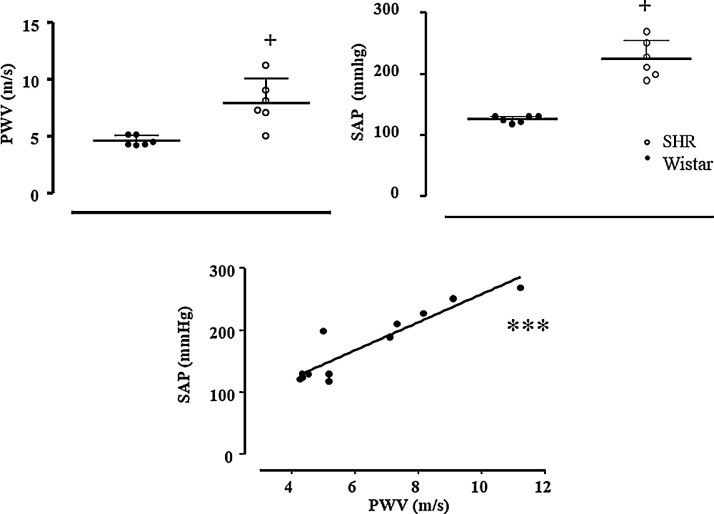
Results of estimated pulse wave velocity (PWV, m/s), systolic arterial pressure (SAP) and Pearson´s correlation test between PWV and SAP (r= 0.923, p< 0.0001) of normotensive (Wistar=6) and hypertensive rats (SHR=6). Significance: + *vs* Wistar, Student T-test, p<0.01; Pearson correlation test *** P<0.0001

**Study 3:** This study was intended to verify the ability of pOpet^Ⓡ^1.0 to detect the increased arterial stiffness in hypertensive rats, known to exhibit higher PWV values when compared with normotensive rats. For this study, 06 male Wistar rats and 06 age-matched (5 months) male spontaneously hypertensive rats (SHR) were used. To this end, blood pressure and PWV were measured in the same rats. In summary, after PWV measurement, all rats underwent carotid catheterization surgery and 24h later, blood pressure was recorded, as published [19]. As shown in Fig. 7, SHR when compared with normotensive Wistar rats, exhibited both elevated systolic arterial pressure (224 ± 13 *vs* 126 ± 2 mmHg, respectively, p=0.0006) and higher PWV estimated values (7.98 ± 0.85 *vs* 4.64 ± 0.17 m/s, SHR *vs* Wistar, respectively, p=0.0032). In addition, PWV estimated values were positively correlated with systolic arterial pressure (r= 0.923, p<0.0001, bottom panel of Fig. 7), confirming that this new noninvasive device for small animals was able to detect age and hypertension-related changes in arteries, therefore estimating different arterial stiffness patterns.

## Conclusion

In conclusion, this present study revealed that: i) estimations of PWV, obtained through the new pOpet^Ⓡ^ 1.0 noninvasive device for small animals, are reproducible, exhibiting great stability; ii) PWV assessments correlate with age-induced changes in arteries’ structure known to increase vascular stiffness; iii) the new pOpet^Ⓡ^ 1.0 system detects different TT assessed in normotensive and hypertensive rats, being a valuable device to estimate arterial stiffness. Therefore, we propose that the noninvasive pOpet^Ⓡ^ 1.0 system for small animals is suitable to estimate *in vivo* PWV in rats. It is a valuable tool that should be used for advancing our understanding on the mechanisms responsible for the control of arterial stiffness, an important predictor of cardiovascular events.

## References

[1] Mitchell G.F., Hwang S.J., Vasan R.S., Larson M.G., Pencina M.J., Hamburg N.M., Vita J.A., Levy D., Benjamin E.J. (2010). Arterial stiffness and cardiovascular events: the Framingham Heart Study. Circulation.

[2] Scuteri A., Morrell C.H., Fegatelli D.A., Fiorillo E., Delitala A., Orru M., Marongiu M., Schlessinger D., Cucca F. (2019). Arterial stiffness and multiple organ damage: a longitudinal study in population. Aging clinical and experimental research.

[3] Nilsson Wadstrom B., Fatehali A.H., Engstrom G., Nilsson P.M. (2019). A Vascular Aging Index as Independent Predictor of Cardiovascular Events and Total Mortality in an Elderly Urban Population. Angiology.

[4] Laurent S., Cockcroft J., Van Bortel L., Boutouyrie P., Giannattasio C., Hayoz D., Pannier B., Vlachopoulos C., Wilkinson I., Struijker-Boudier H. (2006). Expert consensus document on arterial stiffness: methodological issues and clinical applications. European heart journal.

[5] Lacolley P., Regnault V., Segers P., Laurent S. (2017). Vascular Smooth Muscle Cells and Arterial Stiffening: Relevance in Development, Aging, and Disease. Physiological reviews.

[6] Alivon M., Vo-Duc Phuong T., Vignon V., Bozec E., Khettab H., Hanon O., Briet M., Halimi J.M., Hallab M., Plichart M., Mohammedi K., Marre M., Boutouyrie P., Laurent S. (2015). A novel device for measuring arterial stiffness using finger-toe pulse wave velocity: Validation study of the pOpmetre(R). Archives of cardiovascular diseases.

[7] Avolio A.P., Chen S.G., Wang R.P., Zhang C.L., Li M.F., O'Rourke M.F. (1983). Effects of aging on changing arterial compliance and left ventricular load in a northern Chinese urban community. Circulation.

[8] Safar M., Duriez M., Corman B., Levy B. (2001). Endothelium-dependent changes in arterial diameter in old normotensive rats. Clinical and experimental pharmacology & physiology.

[9] Benetos A., Waeber B., Izzo J., Mitchell G., Resnick L., Asmar R., Safar M. (2002). Influence of age, risk factors, and cardiovascular and renal disease on arterial stiffness: clinical applications. American journal of hypertension.

[10] Mattace-Raso F H.A., Verwoert GC, Wittemana JC, Wilkinson I, Cockcroft J, McEniery C, Yasmin, Laurent S, Boutouyrie P, Bozec E, Hansen TW, Torp-Pedersen C, Ibsen H, Jeppesen J, Vermeersch SJ, Rietzschel E, De Buyzere M, Gillebert TC, Van Bortel L, Segers P, Vlachopoulos C, Aznaouridis C, Stefanadis C, Benetos A, Labat C, Lacolley P, Stehouwer C, Nijpels G, Dekker JM, Stehouwer C, Ferreira I, Twisk JW, Czernichow S, Galan P, Hercberg S, Pannier B, Guérin A, London G, Cruickshank JK, Anderson SG, Paini A, Agabiti Rosei E, Muiesan ML, Salvetti M, Filipovsky J, Seidlerova J, Dolejsova M. (2010). Determinants of pulse wave velocity in healthy people and in the presence of cardiovascular risk factors: 'establishing normal and reference values'. European heart journal.

[11] Lindesay G., Ragonnet C., Chimenti S., Villeneuve N., Vayssettes-Courchay C. (2016). Age and hypertension strongly induce aortic stiffening in rats at basal and matched blood pressure levels. Physiological reports.

[12] Morgan E.E., Casabianca A.B., Khouri S.J., Kalinoski A.L. (2014). In vivo assessment of arterial stiffness in the isoflurane anesthetized spontaneously hypertensive rat. Cardiovascular ultrasound.

[13] Beardsley C., Egerton T., Skinner B. (2016). Test-re-test reliability and inter-rater reliability of a digital pelvic inclinometer in young, healthy males and females. PeerJ.

[14] Koo T.K., Li M.Y. (2016). A Guideline of Selecting and Reporting Intraclass Correlation Coefficients for Reliability Research. Journal of chiropractic medicine.

[15] Jordao M.T., Ladd F.V., Coppi A.A., Chopard R.P., Michelini L.C. (2011). Exercise training restores hypertension-induced changes in the elastic tissue of the thoracic aorta. Journal of vascular research.

[16] Amaral S.L., Zorn T.M., Michelini L.C. (2000). Exercise training normalizes wall-to-lumen ratio of the gracilis muscle arterioles and reduces pressure in spontaneously hypertensive rats. Journal of hypertension.

[17] Lindesay G., Bezie Y., Ragonnet C., Duchatelle V., Dharmasena C., Villeneuve N., Vayssettes-Courchay C. (2018). Differential Stiffening between the Abdominal and Thoracic Aorta: Effect of Salt Loading in Stroke-Prone Hypertensive Rats. Journal of vascular research.

[18] Barao F.T.F., Barao V.H.P., Gornati V.C., Silvestre G.C.R., Silva A.Q., Lacchini S., de Castro M.M., De Luccia N., da Silva E.S. (2019). Study of the Biomechanical and Histological Properties of the Abdominal Aorta of Diabetic Rats Exposed to Cigarette Smoke. Journal of vascular research.

[19] Herrera N.A., Jesus I., Shinohara A.L., Dionisio T.J., Santos C.F., Amaral S.L. (2016). Exercise training attenuates dexamethasone-induced hypertension by improving autonomic balance to the heart, sympathetic vascular modulation and skeletal muscle microcirculation. Journal of hypertension.

